# Statement of retraction: Long non-coding RNA LINC01133 silencing exerts antioncogenic effect in pancreatic cancer through the methylation of DKK1 promoter and the activation of Wnt signaling pathway

**DOI:** 10.1080/15384047.2026.2740282

**Published:** 2026-09-29

We, the Editors and Publisher of *Cancer Biology & Therapy*, have retracted the following article:

Weng, Y. C., Ma, J., Zhang, J., & Wang, J. C. (2019). Long non-coding RNA LINC01133 silencing exerts antioncogenic effect in pancreatic cancer through the methylation of DKK1 promoter and the activation of Wnt signaling pathway. *Cancer Biology & Therapy*, *20*(3), 368–380. https://doi.org/10.1080/15384047.2018.1529110

Following publication, concerns were raised by a third party regarding the image integrity of Western Blot Figures 2E and 3 C.

Further investigation by the Publisher additionally identified concerns regarding the qRT-PCR methodology and results described in the article. When approached for an explanation, the authors did not respond to our queries.

As verifying the validity of published work is core to the integrity of the scholarly record, we are therefore retracting the article. The corresponding author has been informed.

We have been informed in our decision-making by our editorial policies and the COPE guidelines.

The retracted article will remain online to maintain the scholarly record, but it will be digitally watermarked on each page as ‘Retracted’.

